# Literature in Plague Time

**Published:** 1889-02-23

**Authors:** 


### Literature in Plague Time.

*(Continued from page 310.)*

Mendicus made what we should now vulgarly term a bad shot when he offered to repeat his prayer in Latin, for though Civis is a good-natured fellow, ready to help him for no other reason than that he is poor and hungry, Uxor, his wife, is a dame of theological proclivities, soundly but not charitably Protestant, who rather doubts the beggar's worth, even when he has told her a long story of his losses in his native Redesdale, where the invading Scots had come and "harried his goods." Hearing his north-country accent, she gives it as her opinion that he is a Scot himself, but is earnestly assured that he hates his northern neighbours so much that he would rather be hanged in a cow-tail than be a "row-footed Scot." The citizen and his wife being moved to charity, Mendicus becomes garrulous, and tells them how on his way to the city he met with waggons and carts filled with children, who were being sent into the country by their parents for fear of infection. Civis has sent his children away, though he himself is as yet resolved to stay in town, look after his business, and risk the possibility of being plague-stricken. "Youth," he says, "are apte to take the plague." He adds to this the true, sad reflection, "And, further, parentes are more naturall to their children than children to their fathers and mothers. Nature doth descende, but not ascende."

Mendicus is not much moved by the plague. It is an ill wind that blows nobody good, he thinks; and alms are more plentiful in plague time, besides the reversionary chances of dead men's shoes and other belongings that come to beggars then. So he goes off cheerily to the house of one Antonius, who is reported to be attacked with the disease, and who has just sent for the Medicus, the renowned Dr. Tocrub.

We are now introduced into Antonius's sick-room, where a long conversation goes on between him and Medicus—a conversation little likely to take place in face of the fact that the doctor at once tells his patient that his sickness is likely to be deadly. Men who have just heard their doom pronounced are little inclined to talk theology and philosophy, especially such men as Antonius; for he is a very evil man. He is a usurer by trade, and has become rich by the misfortunes of others; he is, by his own confession, immoral in life and coarse in conversation; he is, of course, both covetous and dishonest; the little Scripture that he knows fell upon his ears by chance when he had entered a church for the purpose of arresting a debtor. All this he mentions with the frankness of a man who does not know good from evil; but he has his redeeming points. Medicus, with great candour, tells him that he is at the point of death; yet Antonius, the selfish usurer, who is at that moment suffering the direst torments, can so far rise above these and his past self as to enter into a long philosophical discourse with his doctor on Aristotle's views of the soul and its virtues, and afterwards ask what are the best medicines for the prevention and cure of the plague that is killing him, because, he says, "though it help not me, yet I trust it shall do good to others when I am gone."

Perhaps it is unexpected goodness in Antonius that gives opportunity for the publication of these prescriptions; but perhaps it is the author's imperfect art that forces in the information he wants to give in this unlikely way. We all know how the amateur novelist, the writer of a "story with a purpose," works. He wants to give information on archæology, or cookery, or some other science; so, after a very feeble preamble, the Telemachus of the tale makes some remark which gives the mentor occasion for a long homily on the chosen subject. Have we not all read "Sandford and Merton" in our youth, and watched the amiable Harry make opportunity for the discourses of the all-wise Mr. Barlow?

*(To be continued.)*

